# Integrative metagenomic analysis reveals distinct gut microbial signatures related to obesity

**DOI:** 10.1186/s12866-024-03278-5

**Published:** 2024-04-05

**Authors:** Xinliang Hu, Chong Yu, Yuting He, Songling Zhu, Shuang Wang, Ziqiong Xu, Shaohui You, Yanlei Jiao, Shu-Lin Liu, Hongxia Bao

**Affiliations:** 1 Genomics Research Center, Key Laboratory of Gut Microbiota and Pharmacogenomics of Heilongjiang Province, State-Province Key Laboratory of Biomedicine-Pharmaceutics of China, College of Pharmacy, Harbin Medical University, Harbin, China; 2 Department of Biopharmaceutical Sciences (State-Province Key Laboratories of Biomedicine-Pharmaceutics of China), College of Pharmacy, Harbin Medical University, Harbin, China; 3 Harbin Medical University-University of Calgary Cumming School of Medicine Centre for Infection and Genomics, Harbin Medical University, Harbin, China

**Keywords:** Obesity, Gut microbiota, Metagenomics, Microbiome, Virome

## Abstract

Obesity is a metabolic disorder closely associated with profound alterations in gut microbial composition. However, the dynamics of species composition and functional changes in the gut microbiome in obesity remain to be comprehensively investigated. In this study, we conducted a meta-analysis of metagenomic sequencing data from both obese and non-obese individuals across multiple cohorts, totaling 1351 fecal metagenomes. Our results demonstrate a significant decrease in both the richness and diversity of the gut bacteriome and virome in obese patients. We identified 38 bacterial species including *Eubacterium* sp. CAG:274, *Ruminococcus gnavus*, *Eubacterium eligens* and *Akkermansia muciniphila,* and 1 archaeal species, *Methanobrevibacter smithii*, that were significantly altered in obesity. Additionally, we observed altered abundance of five viral families: *Mesyanzhinovviridae*, *Chaseviridae*, *Salasmaviridae*, *Drexlerviridae*, and *Casjensviridae*. Functional analysis of the gut microbiome indicated distinct signatures associated to obesity and identified *Ruminococcus gnavus* as the primary driver for function enrichment in obesity, and *Methanobrevibacter smithii*, *Akkermansia muciniphila*, *Ruminococcus bicirculans*, and *Eubacterium siraeum* as functional drivers in the healthy control group. Additionally, our results suggest that antibiotic resistance genes and bacterial virulence factors may influence the development of obesity. Finally, we demonstrated that gut vOTUs achieved a diagnostic accuracy with an optimal area under the curve of 0.766 for distinguishing obesity from healthy controls. Our findings offer comprehensive and generalizable insights into the gut bacteriome and virome features associated with obesity, with the potential to guide the development of microbiome-based diagnostics.

**Supplementary Information:**

The online version contains supplementary material available at 10.1186/s12866-024-03278-5.

## Introduction

In recent years, the number of obese people has been steadily increasing worldwide, making obesity a global public health issue [1]. According to the World Health Organization (2024), the global number of obese adults has exceeded 890 million and continues to rise [2]. Studies have demonstrated that obesity is linked to numerous complications, including diabetes, cardiovascular disease, cancer, neurological conditions, respiratory disorders, as well as diseases affecting the immune and digestive systems [3].

The human gut microbiome is a complex ecosystem predominantly composed of bacteria, along with small numbers of archaea, viruses, and fungi, which have not been extensively studied, especially in terms of functions [4]. The gut microbiota performs a crucial role in breaking down and fermenting the indigestible carbohydrates, which results in the production of physiologically active substances, including short-chain fatty acids (SCFAs), amino acids and essential vitamins [5]. A growing body of evidence suggests that the gut microbiota significantly contributes to host physiology. Dysbiosis of the intestinal microbiota may contribute to the development of various diseases, including IBD, cancer, diabetes and obesity [6].

Numerous studies have documented significant differences in the composition and abundance of gut microbiota between obese humans and mice in comparison with their respective lean controls [7–10]. Certain specific genera or species have been identified as obesity-associated (e.g., *Corpococcus*, *Clostridium leptum*) or lean-associated (e.g., *Akkermansia*, *Bacteroides*) [11]. Specifically, studies have reported a reduction in the abundance of the *Bacteroidetes* phylum and an increase in the *Firmicutes* phylum in obese individuals compared to their lean counterparts [12]. However, it is worth noting that the specific microbial taxa showing significant differences in relation to obesity have been found to vary across different studies. These inconsistencies can be attributed to variations among studies, including differences in methodologies, sample sizes, populations studied, and the use of different technical platforms. Performing integrated microbiome data analysis across multiple studies using meta-analysis techniques can effectively mitigate the risk of false positives and false negatives [13]. Numerous cross-cohort meta-analyses of obesity-related metagenomics data have been conducted, primarily using 16S sequencing data. However, these analyses have been limited in their ability to comprehensively assess the functional aspects of the gut microbiome [14–16]. The gut virome is also believed to be closely associated with the pathogenesis of several host diseases, including obesity [17–20]. However, the relationship between the gut virome and obesity has not been well-investigated.

To comprehensively characterize the composition and functional features of the gut microbiome in obese populations, we conducted an extensive and in-depth meta-analysis across seven studies. We included 1351 fecal shotgun metagenomics sequencing files from five different countries in this multi-cohort investigation. Incorporating a broader range of obese and non-obese samples, we aimed to accurately analyze shifts in bacterial and viral phylogenetic composition, metabolic functions within the gut microbiome, and correlations between resistance genes, virulence factors, and obesity status.

## Methods

### Data collection

We used PubMed to search for studies that published fecal shotgun metagenomic data of human obesity patients and healthy controls. As search term, we used “(Obesity) AND (Metagenomic)” and collected studies published from year 2012 to 2021. Raw SRA files were downloaded for the included studies from NCBI database. In this meta-analysis, 1351 stool metagenomic sequencing files from 862 subjects along with corresponding metadata including sex, age, nationality, and BMI values were retrieved from NCBI database. These data were derived from seven different published studies including samples from China, Australia, Denmark, Spain and Sweden (Table S1) [7–10, 21–23]. Four of the studies involving Chinese and Danish populations were related to obesity, while the remaining three studies were related to other diseases, and data from these three studies have been used in other metagenomic multicohort analyses [24]. We extracted only healthy control samples from these non-obesity studies and regrouped them according to BMI values. Considering the different body fat characteristics of Asian and European populations, we reclassified the obese and control populations according to the following criteria: for the Chinese population when BMI ≥ 28 was designated as the obese phenotype, BMI < 28 as the control phenotype, and for other countries the grouping criteria were obese samples when BMI ≥ 30 and control samples when BMI < 30 [25, 26].

### Sequence processing and bacterial species diversity analysis

The raw sequencing files were downloaded by SRA Toolkit and split into paired-end FASTQ compressed files for the subsequent analysis. FASTQ C(https://github.com/s-andrews/FastQC) and Fastp [27] were used to remove adapter sequences and low-quality bases (default parameters). Bacterial species relative abundance information was determined using MetaPhlan3.0, a marker gene-based species annotation tool, which is mainly used to analyze the microbial composition of shotgun sequencing metagenomic data [28]. During the annotation process, 14 metagenomic data from the Danish group were failed with annotation due to the too short read length (< 70 bp), and duplicate sequencing results of 6 Spanish populations at different time points were removed in microbiome analysis (but retained during virome mining). Finally, a total of 862 samples were successfully annotated, and the annotation results were used for subsequent analysis. Species α-diversity analysis (Shannon, Simpson, Richness), inter-sample β-diversity analysis (Bray-Curtis) and PCoA (principal co-ordinates analysis) analysis of all samples were done through the vegan R package.

### Differential enrichment analysis of bacterial species

We first performed a multivariate analysis of variance to account for impact of batch effects and potential confounders on the variance analysis. The results showed that the factor from different studies explained 11.796% of the sample variance, followed by nationality explained 10.831% of the variance, and age, gender, and phenotype contributed less to the sample variance. But there is a large overlap between studies and nationalities, so we only consider study factor in the subsequent analysis. Between-cohort and within-study batch effects were alleviated by MMUPHin, an R package developed specifically for microbiome meta-analysis that enables batch processing with covariate control and correction for batch effects [29]. The batch effects were removed using *adjust_batch* function implanted in the R package with factor from different studies as batch factor and phenotype as covariates (parameter setting: batch = “studyID”, covariates = “study_condition”). After batch effect correction, MaAsLin2 (Multivariate Association with Linear Models) was employed to identify differentially enriched bacterial species while adjusting for confounding factors such as age and gender. We retained only the results meeting the criteria of *p* < 0.01, FDR < 0.01, and a prevalence of > 20% [30]. Meanwhile, we also used the linear discriminant analysis effect size (LEfSe) analysis to further validate the differentially enriched species (LDA > 2, *p <* 0.05).

### Bacterial sequence assembly and functional annotation

Metagenomic sequences were assemble into contigs using MEGAHIT (v1.2.9) [31] (parameter setting: --k-min 29 --min-contig-len 1000), and assess the quality of assembly with QUAST(v5.0.2) [32]. The gene prediction was done by Prodigal (v2.6.3) (metagenomic mode) [33]. To construct a non-redundant gene catalogue, we use Cd-hit(Version 4.8.1) [34] to cluster the genes using a sequence identity cut-off of 95% and with a minimum coverage cut-off of 80%. The longest sequence is taken as the representative sequence for further analysis. To estimate the prevalence of non-redundant genes, we first use the BWA tool [35] to re-align these genes to the clean FASTQ files, and then extract the mapping rate and number of aligned reads from the SAM file using SAMtools [36]. The relative gene abundance was obtained by dividing the number of aligned gene reads by the total mapped reads.

The non-redundant gene sets were translated into protein sequences by EMBOSS (v6.6.0.0) [37] and the protein function assignment was carried out by eggNOG-Mapper(EggNOG db 5.0.2) [38]. We extracted KEGG Orthology (KOs) information from the results of functional annotations and determined the relative abundance of KOs after additional processing. Subsequently, we integrated the KOs abundance using PICRUSt2 (specifically, the pathway_pipeline.py) [39] to obtain higher-level abundance information, encompassing Pathways and Modules. The bacterial virulence factors and resistance genes were identified by using Abricate(v1.0.1) (https://github.com/tseemann/abricate) against the Virulence Factor Database(VFDB) and the Comprehensive Antibiotic Resistance Database (CARD) respectively with default parameters. We used FishTaco [40] (parameter setting: -op fishtaco_out_de_novo_inf) to perform the driving species inference analysis, and FishTacoPlot to visualize the results.

### Identification of viral sequences

Viral sequences were recovered from the metagenomic assemblies using Virsorter2(Version 2.2.3) [41] (parameter setting: --include-groups “dsDNAphage, ssDNA” --min-length 5000). CheckV (v0.7.0) [42] was used to evaluated the quality of virus contigs and only kept virus contigs with a completeness greater than 50%. We clustered the vOTUs by Cd-hit on the basis of 95% similarity and 85% coverage, and the longest viral contig was used as the representative sequence of that group. The identified vOTUs were further analyzed by online server PhaBOX with default parameters. PhaBOX is an online server for phage contigs analysis in metagenomic data [43]. For the predicted host bacterial species, we used TaxonKit [44] to obtain their complete lineages and performed statistical analysis at the taxonomic level of the phylum.

### Differential enrichment analysis of virome

To obtain the relative abundance of each vOTUs, the vOTUs contigs were aligned to the clean FASTQ file using bowtie2 (v 2.4.4) [45] (parameter setting: -N 0). The aligned read counts of vOTUs were then extracted from the SAM files by SAMtools and normalized to relative abundance. The vegan R package was used to perform both alpha diversity (Shannon, Simpson, Richness) and beta diversity (Bray-Curtis) analyses of vOTUs, as well as PCoA analyses. To identify differentially enriched vOTUs, we used the LEfSe tool based on the linear discriminant analysis (LDA) algorithm for analysis (LDA > 2, *p* < 0.01).

### Correlation analysis and phenotype prediction

For the correlation analysis between viruses and bacteria, Spearman’s correlation was calculated by Hmisc R package. Only species and phage family with a prevalence greater than 20% were included in the correlation analysis and the BH (Benjamini & Hochberg) method was used for *P*-value correction to ensure the reliability of the results. The Shannon index correlation and richness correlation between the microbiome and virome was performed using the glm() function in the R.

The SIAMCAT [46] (Statistical Inference of Associations between Microbial Communities And host phenoTypes) R toolkit was used to explore the association between the species and functional characteristics of the gut microbiome and obesity phenotypes. In this study, we mainly used the LASSO algorithm provided by SIAMCAT for modeling. We firstly built a prediction model based on the relative abundance of bacterial species (or vOTUs) or the relative abundance of KOs, and then built a hybrid prediction model based on both datasets. The cross-validation adopts the method of 10 folds and 10 crosses. The filtering threshold of the relative abundance of features was 1 × 10^−5^, and the model evaluation was done through the ROC and PROC curves.

## Results

### Taxonomic characterization of the gut bacteriome

**Fig. 1:**
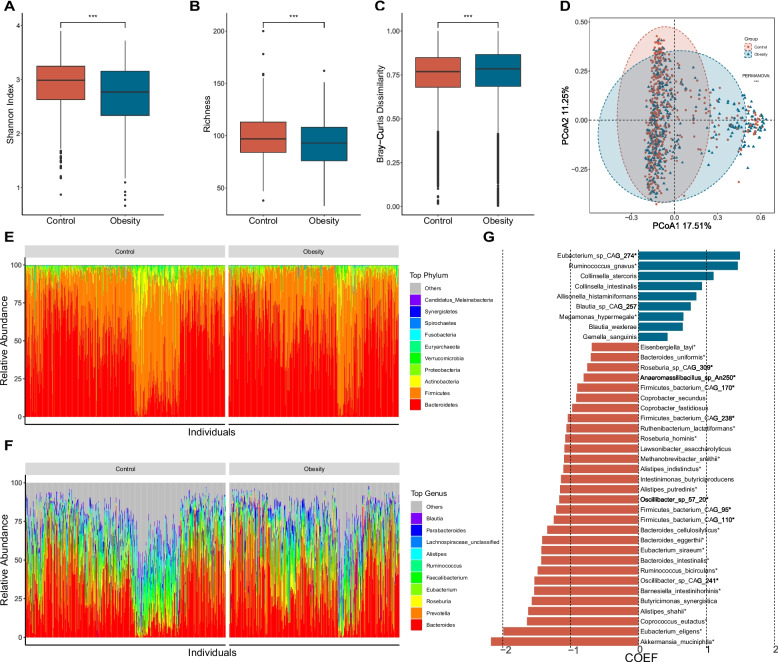
Structure and differential enrichment analysis of gut microbial communities in obesity. **A**, **B**, **C** Comparison of α diversity (Shannon index; **A**), β diversity (Bray-Curtis similarity index; **B**) and gene richness(**C**) of microbial content between the obesity and the control group. **D** PCoA analysis based on Bray-Curtis distance, with PERMANOVA used to assess differences in bacterial composition between the obesity and control groups. **E** Relative abundance of the top 10 bacterial phyla. **F** Relative abundance of the top 10 bacterial genera. **G** Differentially enriched species obtained through MAssLin2 analysis (*p* < 0.01, FDR < 0.01), with bacterial species names marked with “*” indicating consistency with LEfSe analysis results (LDA > 2, *p* < 0.05). The x axis represents the coefficient value calculated by MaAsLin2 analysis

After eliminating duplicate and low-quality samples, our analysis dataset included a total of 396 obese samples and 466 control samples from seven independent studies (Table S1). The high-quality reads that remained after screening with Fastp were aligned using Metaphlan 3.0. This alignment revealed that bacteria accounted for the majority (98.80%) of the total samples, with a small percentage of archaea (0.53%) identified (referred to as the bacteriome here). The species level biodiversity analysis of the gut microbiota showed that the α-diversity (Shannon Index, *p* < 0.001) and richness of the control group were significantly higher than those of the obesity group (Fig. 1A,B). Furthermore, the β-diversity of the obese group (Bray-Curtis, *p* < 0.001) was significantly higher than that of the healthy control group (Fig. 1C). To delve deeper into the differences in the gut microbiome, we conducted a principal-coordinate analysis (PCoA). The results of the PCoA exhibited a distinct separation between the two groups (PERMANOVA, *p* < 0.001) (Fig. 1D), highlighting significant disparities in their microbial compositions.

PERMANOVA (Permutational multivariate analysis of variance) was employed to examine the factors contributing to sample differences. In this study, we primarily focused on disease phenotype, age, sex, country, and study as the main influencing factors. Our analysis indicated that the ‘phenotype’ factor of the participants accounted for a small portion of the variation, which aligns with previous studies [24]. Country and batch factors explained a significant portion of the variation, but the confounder analysis by SIAMCAT showed a substantial overlap between these two factors (Fig. S1A). So, we considered only the batch factors in subsequent analyses. Initially, we corrected the batch effects using MMUPHin and then employed MaAsLin2 to identify differential species. We included the two factors, sex ratio (MF) and age, as covariates in our analysis. After correcting for batch effects, the contribution of batch factors to the sample variance was reduced from 11.796 to 4.852%, and the correction effect was significant (Fig. S1B). We only retained the results from the differential enrichment analysis that met the criteria of *p* < 0.01 and FDR < 0.01, with a prevalence cutoff > 20%.

Microbial compositions analysis identified the *Bacteroidetes*, *Firmicutes*, *Actinobacteria*, *Proteobacteria* and *Verrucumicrobia* as the most dominant phyla in both groups (Fig. 1E). Further phylum level study revealed that the obesity group exhibited markedly higher levels of *Bacteroidetes*, *Ascomycota* and *Fusobacteria Ascomycota* and lower levels of *Actinobacteria*, *Verrucomicrobia*, *Firmicutes*, *Synergistetes* and *Euryarchaeota* than those of the control groups (Fig. 1E, Fig. S2A). At the same time, the average abundance ratio of *Firmicutes* to *Bacteroides* in the obese group was significantly lower than that in the control group (Fig. S2B). This observation contrasts with the findings of certain previous studies [47]**.** At the genus level, the relative abundance of 25 bacterial genera and two archaeal genera was significantly different between the two groups (Fig. 1F, Fig. S2C). We then conducted a species-level comparison of the gut microbiome and identified 39 species that exhibited significant differences in relative abundance between the two groups. Among these, 29 bacterial species including *Akkermansia muciniphila*, *Eubacterium eligens*, *Coprococcus eutactus*, and *Alistipes shahii*, and one archaeal species (*Methanobrevibacter smithii*), were found to be significantly enriched in the control group. In contrast, 9 bacterial species, including *Eubacterium* sp. CAG:274, *Ruminococcus gnavus*, *Collinsella stercoris*, and *Megasphaera elsdenii*, were enriched in the obese group (Fig. 1G). We confirmed the differential enrichment of species in both groups through LEfSe analysis, revealing that 3 species in the obesity group and 25 species in the control group exhibited significant differential enrichment (Fig. 1G).

### Taxonomic annotation and comparison of gut virome

**Fig. 2:**
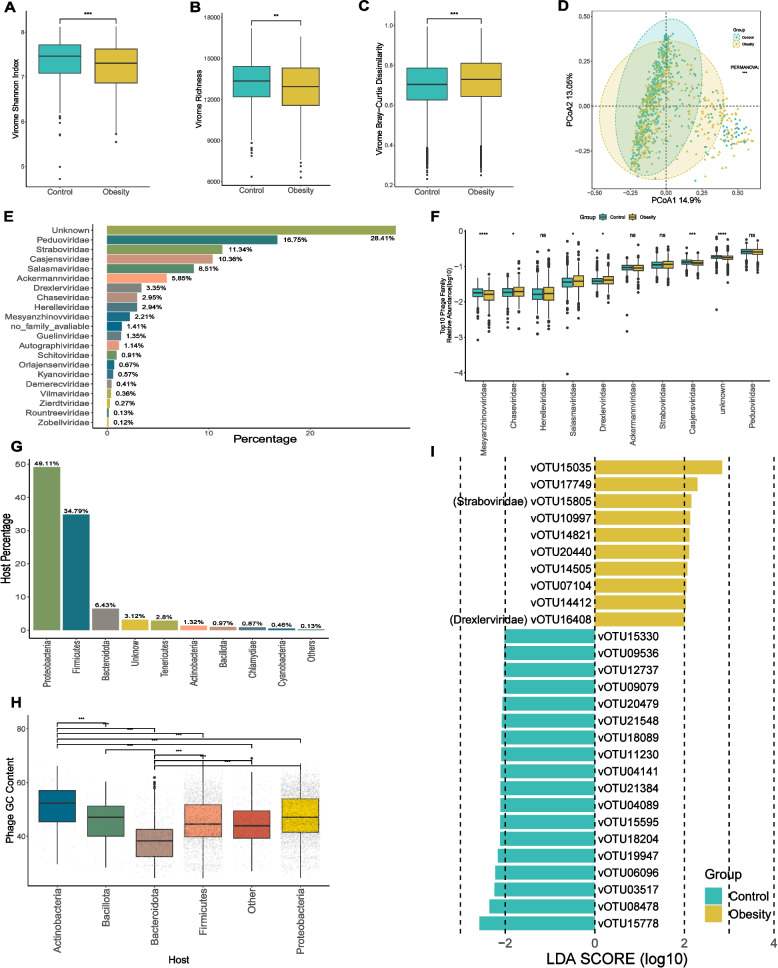
Characteristics of the gut virus catalogue and gut viral communities in obesity. **A**, **B**, **C** Comparison of α diversity (Shannon index; **A**, gene richness **B**) and β diversity (Bray-Curtis similarity index **C**) of viral community between the obesity and the control group. **D** PCoA analysis based on Bray-Curtis distance, with PERMANOVA used to assess differences in viral composition between the obesity and control groups. **E** Percentage of different viral families in the virome. **F** Relative abundance of the top 10 viral families. **G** Percentage of different phage hosts in the overall composition. **H** Boxplot of the GC content of phages. The x-axis representing the corresponding hosts at the phylum level. The x-axis representing the phage hosts at the phylum level. (I) vOTUs differentially enriched between the two groups obtained through LEfSe analysis (LDA > 2, *p* < 0.01)

Phages, or bacteriophages, are viruses that infect bacteria and have the potential to modulate the structure of the human gut microbiome by lysing bacterial hosts and facilitating horizontal gene transfer [48, 49]. Numerous studies have suggested a strong correlation between the viral component of the gut microbiome and obesity. To further investigate this relationship, we assessed the viral component using the virus identification tool Virsorter2. We finally obtained 27,651 high-quality viral contigs, which were then clustered into 22,620 representative viral operational taxonomic units (vOTUs). To understand the intra-community and inter-community diversity of the virome in the two groups, we analyzed the α-diversity, richness, and β-diversity. The analysis of gut viral composition revealed a significant decrease in both viral α-diversity (Shannon Index, *p* < 0.001) and richness in the obese group, and significant increase in β-diversity by comparing with the control group (Bray-Curtis, *p* < 0.001) (Fig. 2A, B, C). Bray-Curtis distance-based PCoA analysis showed that the obese and control groups were significantly divided into two distinct clusters (PERMANOVA, *p* < 0.001) (Fig. 2D). These results indicate that the gut virome profiles of subjects in the obese group differ significantly from those in the healthy control group. In our study, out of 22,620 vOTUs, 11,290 were finally annotated as phages by PhaBOX. Among these, 70.18% were taxonomically assigned to 19 viral families, with *Peduoviridae* being the most abundant family (16.75%) (Fig. 2E). Among the top 10 phage families with the highest average relative abundance, *Mesyanzhinovviridae*, *Chaseviridae*, *Salasmaviridae*, *Drexlerviridae*, and *Casjensviridae* showed significant differences in abundance between the obese and control groups (Fig. 2F). To further predict the lifestyle of these phages, the phage genomes or contigs are classified via PhaTYP, a tool combined in PhaBOX. The predictive outcome indicates that 58.70% of the phages were identified as temperate phages, while 41.30% were classified as lytic phages (Table S2). Furthermore, the most prevalent identifiable hosts at the phylum level are primarily *Proteobacteria* (49.11%), *Firmicutes* (34.79%), and *Bacteroidota* (6.43%) (Fig. 2G). Other phyla, such as *Tenericutes*, *Actinobacteria*, *Bacillota*, *Chlamydiae*, and *Cyanobacteria*, were also represented (Fig. 2G). In addition, we analyzed the genomic GC content and size of phages associated with these hosts. Our findings showed that phages with *Bacteroidota* as their host exhibit significantly lower GC contents and larger genomic sizes compared to other phages (Fig. 2H, S2D). Utilizing the relative abundance data of vOTUs, we conducted a differential enrichment analysis employing LEfSe. This analysis revealed 18 vOTUs that exhibited depletion in obesity and 10 vOTUs that displayed enrichment in obesity (LDA > 2.0, *p* < 0.01). Notably, out of these, only two vOTUs, namely vOTU15805 (*Straboviridae*) and vOTU16408 (*Drexlerviridae*), were classified within known phage families (Fig. 2I).

### Functional alternation of gut microbiome in obesity

**Fig. 3:**
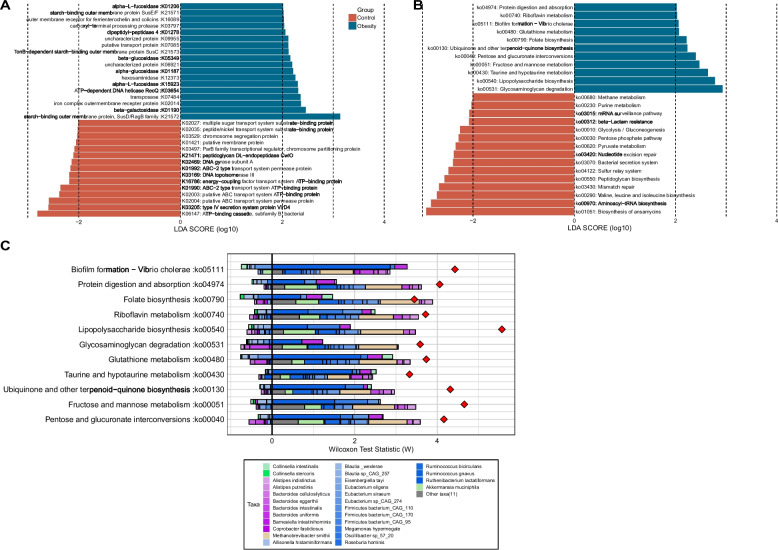
The microbiota functional characterization in obesity. Differential enrichment analysis of the KOs (**A**) and KEGG pathways (**B**) between the obesity and control groups. KOs or KEGG pathways with LDA > 2.0 and *p* < 0.05 are shown. Blue and red color represent obesity- and control- enriched KOs or pathways, respectively. (**C**) Taxon-level contribution profiles of the functional shift in the obesity. The x-axis depicts the ranking and statistical scores, while the y-axis represents the associated pathways. Taxa attenuating each functional shift are presented on the left side of the vertical line, whereas those driving each functional shift are depicted on the right side of the vertical line. For each KEGG pathway, the top bars represent contributions from obesity-associated taxa and the lower bars represents contributions from obesity-depleted taxa. Red diamonds represent taxa-based functional shift scores

To gain functional insights into the gut microbiome, we used eggNOG-Mapper for KEGG functional annotation at the assembly level. This annotation assigned genes to 10,503 KEGG orthologs (KOs), with 33 of them exhibiting differential abundance between the obesity and control groups (LDA > 2.0, *p* < 0.05) (Fig. 3A). Among them, 18 KOs are significantly enriched in the obesity group, including digestive enzyme-coding genes like beta-galactosidase (K01190), fucosidase (K15923, K01206), glucosidase (K01187, K05349), and starch utilization system proteins (K21571, K21573, K21572). Conversely, 15 KOs are significantly enriched in the control group, encompassing genes encoding ABC transporter systems (K06147, K02004, K02003, K01990, K01992, K16786, K02027), type IV secretion system protein VirD4 (K03205), and DNA topoisomerase III (K03169) (LDA > 2, *p* < 0.05, Fig. 3A). Subsequently, the differentially enriched KEGG pathways and modules were identified (Fig. 3B, S3A). We revealed significant enrichment of 11 metabolic pathways and depletion of 15 metabolic pathways in the obesity group (Fig. 3B). The representative obesity enriched pathways included the Glycosaminoglycan degradation pathway (ko00531), Fructose and mannose metabolism (ko00051), Protein digestion and absorption (ko04974) and Lipopolysaccharide (LPS) biosynthesis pathways (ko00540) etc. (Fig. 3B). Moreover, the pathways associated with cofactors and vitamins metabolism or biosynthesis, such as Riboflavin metabolism, Folate biosynthesis and Ubiquinone and other terpenoid-quinone biosynthesis, were also significantly enriched in obesity group (Fig. 3B). We also discovered 15 metabolic pathways that were diminished in the obese group. Among these, the most noteworthy was the biosynthesis of ansamycins, a pathway related to terpenoids and polyketides metabolism. Additionally, pathways related to carbohydrate metabolism, including glycolysis/gluconeogenesis, pentose phosphate and pyruvate metabolism, were enriched in the control group. Notably, the bacterial secretion system was found to be depleted in the obese group, which is of particular interest (Fig. 3B).

To identify the individual taxa that made the greatest contribution to the functional shifts, we used FishTaco, a permutation-based method for further analysis. Unlike the simple Sperman correlation analysis, FishTaco integrates both taxonomic and functional comparative information, which offer a more comprehensive, dynamic, and quantitative analysis of microbial contributions to pathways. Remarkably, the differentially abundant metabolic pathways identified between the obesity and control groups exhibited a high degree of concordance with the results obtained from the KEGG pathway-level enrichment analysis. Among all the significantly enriched functions observed in obesity, *Ruminococcus gnavus* emerges as the primary driver (Fig. 3C). In contrast, within the pathways enriched in the control group, the shifts in functionality appear to be attributed to fluctuations in the abundances of *Methanobrevibacter smithii*, *Akkermansia muciniphila*, *Ruminococcus bicirculans*, and *Eubacterium siraeum* (Fig. 3C, S3B). The most significant obesity-enriched glycosaminoglycan degradation pathway (ko00531) driver species were *Bacteroides uniformis* and *Ruminococcus gnavus*, whereas *Eubacterium* sp. CAG:274, *Blautia wexlerae* and *Blautia* sp. CAG:257 may inhibit this functional shift. The control-enriched “biosynthesis of ansamycins” pathway was driven mostly by *Methanobrevibacter smithii*, *Akkermansia muciniphila, Ruminococcus bicirculans*, *Firmicutes bacterium* CAG:170, *Eubacterium siraeum* and *Firmicutes bacterium* CAG:110, while *Bacteroides intestinalis*, *Firmicutes bacterium* CAG:95 may play an inhibitory role (Fig. S3B).

### Bacterial virulence factors and resistance gene analysis

**Fig. 4:**
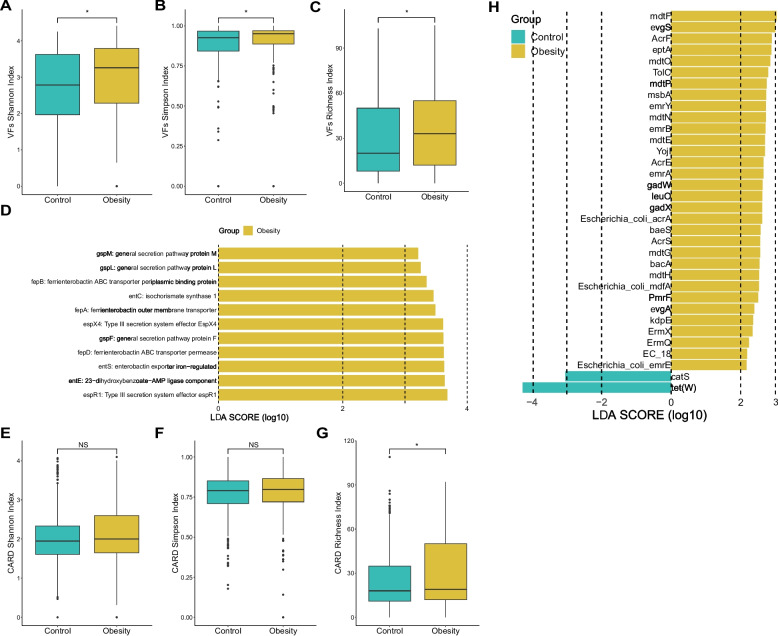
Bacterial virulence factor and resistance gene annotation. **A**, **B**, **C** Comparison of bacterial virulence factor Shannon index (**A**), Simpson index (**B**), and richness (**C**) between the obesity and control groups. (**D**) Differential enrichment analysis of bacterial virulence factors (LEfSe, LDA > 2.0, *p* < 0.01). The yellow bar represents the obesity enriched VFs. **E**, **F**, **G** Box plots depicting bacterial resistance gene Shannon index (**E**), Simpson index (**F**), and richness (**G**). **H** Differential enrichment analysis of bacterial resistance genes (LDA > 2.0, *p* < 0.001). The yellow bar represents the obesity enriched VFs and the green bar represent the VFs enriched in control group

Elevated carriage of virulence factors (VFs) and antibiotic resistance genes (ARGs) by the gut microbiome has been associated with various diseases, including obesity [50, 51]. Bacterial VFs and ARGs were identified by screening the assembled sequences using ABRicate. In total, 218 VFs were identified across both groups (Table S3). Notably, the α-diversity (measured by Shannon and Simpson indices) and richness of VF-related genes were significantly higher in the obesity group compared to the control (Fig. 4A, B, C). Through logistic regression analysis, we discovered a significant correlation between the richness of bacterial VF-related genes and the obesity phenotype (OR 1.0098, CI1.0032–1.0166, *p* = 0.004). According to the results of a LEfSe analysis, 11 VF-related genes were found to be significantly enriched in the obesity group (Fig. 4D). The representative obesiy-enriched VF-related genes included substrates for type II and type III secretion systems (*gspF, gspM, gspL, espX4, espR1*), genes associated with enterobactin biosynthesis (*entC, entE, entS*), and genes involved in ferric enterobactin transport (*fepA, fepB, fepD*) (LDA > 2.0, *p* < 0.01) (Fig. 4D).

In the antibiotic resistome analysis, a total of 232 ARGs were identified across both groups (Table S3). Subsequent diversity analysis revealed a higher mean value of Shannon and Simpson indices in the obesity group, though these differences were not statistically significant (Fig. 4E, F). However, we observed a significantly higher richness of ARGs in the obesity group compared to the control group (*p* < 0.05) (Fig. 4G). Through application of LEfSe analysis, we pinpointed 32 ARGs that were significantly enriched in the obesity group, notable examples of which include *mdtF*, *evgS*, and *Escherichia coli acrA* (LDA > 2, *p* < 0.001) (Fig. 4H). Moreover, when comparing the abundance of resistance genes in samples from various countries, we observed that samples from China exhibited a significantly higher presence of ARGs compared to the other four countries (Fig. S4).

### Viral-bacterial correlations analysis in obesity

**Fig. 5:**
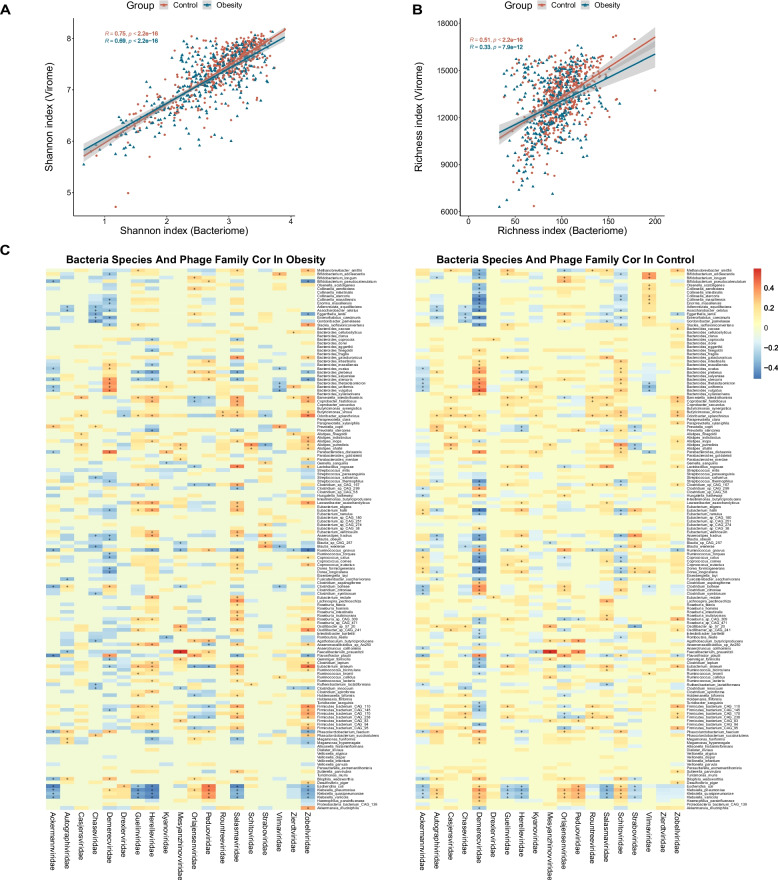
Spearman correlation analysis between bacteria and phage families in the obesity and control groups. **A** Regression analysis between bacterial and viral Shannon indices. **B** Regression analysis between bacterial and viral richness. **C** Transkingdom correlations between the gut virome and bacteriome. The bacterial species with a prevalence of less than 20% were filtered out. Results in the heatmap labeled with “+” indicate r > 0.2 or r < − 0.2. The correlations with *p* < 0.05 and false discovery rate of < 0.05 were regarded as significant and selected for visualization. The viral species are classified by family level in columns and bacterial species are in rows

In order to characterize the association between the gut virome and bacteriome in both obese subjects and healthy controls, a comparative analysis of phage and bacterial profiles within these two groups was conducted. The regression analysis revealed significant positive correlations of the Shannon index and richness between the bacteriome and virome in both the control and obese groups, suggesting a close relationship between the bacterial-viral structures in the human gut (Fig. 5A, B). To further assess the correlation between them, we employed Spearman’s correlation coefficient to analyze the relationship between bacteria species and phage family levels. The results indicated that the number of correlations between the virome and bacteriome notably decreased in the obese group compared to the control group (993 vs. 1130, *p* < 0.001; Fig. 5C). Specifically, the correlations between the virus families *Vilmaviridae* and *Chaseviridae* with their bacterial hosts were significantly stronger in the control group (*p* < 0.01). Conversely, the correlation between the viral families *Zierdtviridae* and *Drexlerviridae* with gut bacteria was notably more intensive in the obese groups (Wilcoxon Test *p* < 0.01, ANOVA Test, *p* < 0.05, Fig. 5C). These results imply the existence of robust and intricate viral-bacterial relationships within the human gut. Furthermore, it raises the possibility that alteration in the virome-bacteriome relationship may be associated with the microbiome dysbiosis in obesity.

### Obesity prediction based on multi-kingdom signatures

**Fig. 6:**
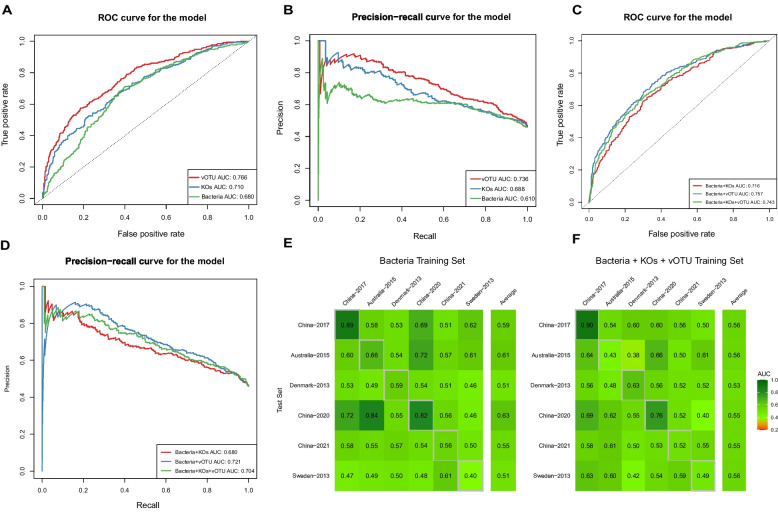
Classification based on species abundance and functional composition for obesity status. **A** ROC curves for the classification models based on taxonomic (microbial species or vOTUs abundance) and functional profiles (KEGG ortholog abundances) of microbiome respectively. **B** PROC curves to evaluate the model’s classification performance based on taxonomic and functional profiles of microbiome respectively. **C** ROC curves for the classification models based on taxonomic (microbial species or vOTUs abundance) and functional profiles (KEGG ortholog abundances) of microbiome in combination. **D** PROC curves to evaluate the model’s classification performance based on taxonomic and functional profiles of microbiome in combination. **E**, **F** Cross-study validation of statistical models trained on bacterial species alone(**E**), or combination of microbial species, vOTUs and KEGG ortholog abundances(**F**)

Finally, we employed the machine learning framework provided by SIAMCAT to assess the predictive potential of species and functional features of gut microbes for obesity. We evaluated the predictive capacity of bacterial species traits, vOTUs traits, and KO functional traits individually. Notably, we observed higher predictive accuracy for vOTUs relative abundance (AUC-ROC 0.766, PRC-ROC 0.736) compared to that of KO functional traits (AUC-ROC 0.710, PRC-ROC 0.688) and bacterial species traits (AUC-ROC 0.680, PRC-ROC 0.610) (Fig. 6A, B). A model trained on the combined relative abundances of differential bacteria, vOTUs, and KOs exhibited higher predictive power compared to using bacterial species or KO functional traits alone (Fig. 6C, D). However, its predictive accuracy remained lower than that achieved using vOTUs alone. To evaluate the influence of different algorithms on prediction accuracy, we reanalyzed the bacteria and vOTUs data using the Enet algorithm, resulting in AUC-ROC values of 0.680 for bacterial data, which were not significantly different from those obtained with the LASSO algorithm. However, the predictive accuracy of vOTUs data decreased (AUC-ROC value = 0.666).

We assessed the generalizability of various research models through cross-study validation, but the AUC-ROC values for these prediction models are generally not high. The AUC-ROC values for predicting obesity based on bacterial species characteristics ranged from 0.40 to 0.84, with prediction accuracy exceeding 0.7 in a few cohorts (Fig. 6E). Among these models, the model based on the cohort ‘China-2020’ can well predict the obesity phenotype in cohort ‘China-2020’ and the cohort ‘Australia− 2015’. Meanwhile, the cohort ‘China-2020’ exhibited the highest average prediction accuracy (AUC-ROC 0.63) (Fig. 6E). The situation is similar for predictive models built with other single data sets. AUC-ROC values using KO data ranged from 0.21 to 0.76 (Fig. S4B), while AUC-ROC values derived from vOTU data ranged from 0.34 to 0.68, and the average AUC-ROC value is less than 0.6 (Fig. S4C). The cross-study validation results of prediction models using combined data sets did not show a significant improvement over those using a single data set. The AUC-ROC values ranges from 0.42 to 0.69，and the average AUC-ROC value is less than 0.6 as well (Fig. 6F). These results suggest that obesity prediction models based on a single cohort do not always apply well to other cohorts, which may reflect the individual specificity of the gut microbiome.

## Discussion

Mounting evidence has highlighted a promising link between the gut bacteriome or virome and obesity [11]. Nevertheless, systematic investigations into the intestinal bacteriome and virome in the context of obesity have been lacking. In this study, we conducted an extensive meta-analysis of metagenomic data from both obese and non-obese control subjects, sourced from public databases. Our findings revealed a significant reduction in both bacteriome and virome diversity and richness in obesity. Furthermore, the cross-kingdom correlations between the bacteriome and virome were found to be diminished in individuals with obesity. We then identified significant metabolic function alterations in the bacterial communities of obese individuals and pinpointed the responsible species driving these shifts. Notably, the analysis of the virulence group revealed significant correlations with obesity, suggesting a non-negligible role of the virulence group in obesity development.

The *Firmicutes* to *Bacteroidetes* (F/B) ratio is one of the parameters used to assess gut microbiota in relation to various diseases, including obesity. Several earlier studies have reported an elevated F/B ratio in the gut microbiota of obese individuals compared to lean counterparts. In fact, the F/B ratio has been proposed as a potential hallmark of obesity in some research. However, in contrast to these findings, a number of studies have reported no alteration or even a decrease in the F/B ratio in the context of obesity [12]. In this study, we identified a decreased F/B ratio in obese patients (Fig. S1B). These variations in results could have arisen from differences in study methodologies and the selection of study subjects. Therefore, we maintain that, at this point, the F/B ratio cannot be considered a reliable marker for obesity.

Our analysis revealed a total of nine bacterial species that were enriched in the obese group. As the species most enriched in obesity, *Eubacterium* sp. CAG:274 is reported here for the first time as an obesity-related bacterium. Notably, *Megamonas hypermegale*, *Ruminococcus gnavus*, *Allisonella histaminiformans, Collinsella stercoris* and *Collinsella intestinalis* are classified as “harmful bacteria” that have been previously demonstrated to be positively correlated with the development of obesity [52–56]. The alignment with prior research reinforces the consistency of our findings. However, the case of *Blautia wexlerae* contradicts previous findings. A prior study indicated a depletion of *Blautia wexlerae* in obese children, which was associated with anti-inflammatory properties [57]. In a recent study, oral administration of *Blautia wexlerae* to mice induced metabolic changes and anti-inflammatory effects, ultimately improving obesity and diabetes [58]. Further work is needed to confirm the role of *Blautia wexlerae* in obesity. Most of the microbes enriched in control group that we identified are known as “beneficial microbes” related to non-obesity or responsible for various anti-inflammatory functions in the gut**.** For example, *Akkermansia muciniphila* is a promising bacterium for modulating obesity, while *Eubacterium eligens* could promote host health by producing anti-inflammatory agents [59, 60]. However, *Alistipes shahii,* previously recorded to be associated with obesity and gut inflammation in Japanese population [61], is significantly enriched in healthy control group in our study, which is in line with other reported studies [62, 63].

Obesity is recognized as a low-grade inflammatory condition contributing to systemic and adipose tissue inflammation [64, 65]. In our functional analysis, we identified significant enrichment of pathways associated with LPS synthesis in the obese group. This finding holds substantial implications, as heightened LPS release from the gut microbiota can lead to elevated circulating LPS levels and the subsequent release of pro-inflammatory factors within the body. These factors collectively contribute to weight gain, adiposity, and insulin resistance, ultimately increasing the risk of conditions like obesity and fatty liver [66]. Furthermore, our analysis revealed the enrichment of various metabolic pathways in the obese group, including riboflavin metabolism, folate biosynthesis, taurine and hypotaurine metabolism, glutathione metabolism, and ubiquinone and other terpenoid-quinone biosynthesis. Among these, taurine and hypotaurine metabolism have been associated with intestinal inflammation due to their potential to dysregulate the gut microbiota [67]. The remaining pathways primarily exhibit antioxidant effects, likely reflecting a response to the elevated oxidative stress levels commonly seen in obesity. Our data also show a decreased microbial capacity for the biosynthesis of ansamycins in obesity, a group of antibiotics displaying anticancer, antibacterial activity [68, 69]. It is intriguing to note that we observed a depletion of the bacterial secretion system pathway and the Type IV secretion system module in the obese group (Fig. S3A). Moreover, a prior study also reported the absence of the Type VI secretion system module in the obese group [7]. Bacterial secretion systems have a significant connection to bacterial competition and their pathogenicity toward the host. Variations in the abundance of these secretion systems within the intestinal flora may indicate shifts in the dynamics of microbial interactions and the overall ecological balance within the gut of obese patients. Moreover, these changes could affect the roles of intestinal bacteria in the overall health of the host.

Our analysis revealed that the richness and α-diversity of the VF-related genes were significantly higher in the obese group compared to the control group. Furthermore, the detection of differentially enriched virulence factor genes, such as *espX4* and *fepA*, was consistent with the findings of previous studies [51]. Numerous studies have also demonstrated an association between virulence factors and inflammatory responses in humans [50], further indicating that the inflammatory conditions observed in obese patients might be attributable to the release of these virulence factors. Many virulence factor-related genes remain unidentified. Thus, it is possible that the distribution of these genes in the gut microbes of obese patients is more extensive than what our study revealed.

The gut virome is increasingly reported to be correlated with different diseases, including inflammatory bowel disease (IBD), cancer, diarrheal diseases and obesity [17]. Our study indicates that the gut virome may play a significant role in obesity, which is consistent with previous literature [19]. In a recent study, researchers found that transplantation of fecal viral-like particles (VLPs) from lean mice to obese mice could lead to a notable reduction in weight gain and alleviated symptoms of type 2 diabetes (T2D) in the recipient obese mice, suggesting a potential contribution of gut virus to obesity development [70]. It is currently understood that a significant portion of the human virome is composed of bacteriophages, while a substantial portion remains unknown [71]. Within our analysis, over half of the vOTUs remained unannotated, while the annotated phages were mainly classified as *Peduoviridae, Straboviridae*, and *Casjensviridae*. To further evaluate the correlation between gut microbes and obesity phenotypes, we constructed predictive models and evaluated the models by machine learning. A recent study using large-scale gut microbiome data for machine learning to explore the relationship between gut microbiota and obesity obtained a strong correlation between them [72]. Nevertheless, that study was restricted to a singular Chinese population and did not encompass an analysis of the viral composition. However, in our cross-study validation, the prediction models showed a poor discriminative ability. The individual characteristics of the gut microbiome are determined by genes, geographical location and lifestyle factors, and so on [73]. A study of populations in different regions of China also suggests that geographical differences may limit the application of metabolic disease diagnostic models based on gut microbes [74]. Although there are still some limitations in building machine learning models to predict obesity phenotypes, relevant methods still have great application potential.

## Conclusion

In summary, we have systematically examined the broad patterns of gut microbiome alterations among individuals with obesity. When compared to healthy counterparts, substantial shifts were observed in the characteristics of the gut microbiome. This was underscored by the presence of 39 distinct bacterial species and 28 vOTUs, accompanied by a noticeable decline in the correlation between the bacteriome and virome. Moreover, our in-depth functional analysis and investigation into the multi-kingdom signature within the context of the obesity-related gut microbiome study further accentuate the potential significance of the gut microbiome as a crucial factor contributing to the obesity phenotype. Our study provides new insights into the understanding of the role of gut microbiome alterations in obesity and may be useful for clinical intervention studies.

### Supplementary Information

**Additional file 1.** Figure S1. Correction of metagenomic data from different cohorts. (A)Assessment of confounders by SIAMCAT. (B) Scatter plot before and after batch effect correction using MMUPHin. Figure(left): Gamma parameter characterizes the mean of each feature by batch. Different batches were marked with different colors. The color points on the figure converge towards 0, indicating an increase in similarity among the parameters. Figure(right): Comparison of the mean of each feature between batch before and after adjustment. A feature is depicted as a pair of interconnected points, with each point representing that feature within a specific batch. Figure S2. Phyla displaying different abundance in obesity and viral component analysis in obesity. (A) Bacteria phylum significantly altered in obesity (p < 0.05). (B) Bacteroidetes/Firmicutes ratio and relative abundance of Bacteroidetes and Firmicutes in obesity and control group (** p < 0.01, *** p < 0.001). (C) Microbial genus significantly altered in obesity (p < 0.001). The blue bar represents obesity and the red bar represents control. (D) Phage genome sizes from different hosts, with the x-axis representing the respective hosts and the y-axis indicating genome size (KB). Figure S3. Functional analysis of the microbiome. (A) Differential enrichment analysis of the KEGG modules between the obesity and control groups (LEfSe, LDA > 2.0, p < 0.001). Blue and red color represent obesity- and control- enriched modules respectively. (B) Taxon-level contribution profiles of the functional shift in control group. The x-axis depicts the ranking and statistical scores, while the y-axis represents the associated pathways. Taxa attenuating each functional shift are presented on the left side of the vertical line, whereas those driving each functional shift are depicted on the right side of the vertical line. For each KEGG pathway, the top bars represent contributions from obesity-associated taxa and the lower bars represents contributions from obesity-depleted taxa. Red diamonds represent taxa-based functional shift scores. Figure S4. The microbial ARGs distribution and cross study validation. (A) Abundance of intestinal ARGs in populations of different countries. (B) Cross-study validation of statistical models trained on functional profiles (KEGG ortholog abundances) alone. (C) Cross-study validation of statistical models trained on vOTUs abundance

**Additional file 2.**

**Additional file 3.**

**Additional file 4.**

## Data Availability

The main code used in this study and the main data generated are available on GitHub (https://github.com/Hxl2023-ZH/Metagenomic-2023).

## Abbreviations

SCFAs: short-chain fatty acids
vOTUs: viral operational taxonomic units
KOs: KEGG orthologs
VFs: virulence factors
ARGs: antibiotic resistance genes
LPS: Lipopolysaccharide
PCoA: principal-coordinate analysis
LEfSe: linear discriminant analysis effect size
