# Supplementary material for: Integrative metagenomic analysis reveals distinct gut microbial signatures related to obesity
Source: BMC Microbiol. 2024 Apr 5;24:119. doi: 10.1186/s12866-024-03278-5 (PMC10996249; doi:10.1186/s12866-024-03278-5)
Supplement: Supplementary file 1 — Additional file 1. Figure S1. Correction of metagenomic data from different cohorts. (A)Assessment of confounders by SIAMCAT. (B) Scatter plot before and after batch effect correction using MMUPHin. Figure(left): Gamma parameter characterizes the mean of each feature by batch. Different batches were marked with different colors. The color points on the figure converge towards 0, indicating an increase in similarity among the parameters. Figure(right): Comparison of the mean of each feature between batch before and after adjustment. A feature is depicted as a pair of interconnected points, with each point representing that feature within a specific batch. Figure S2. Phyla displaying different abundance in obesity and viral component analysis in obesity. (A) Bacteria phylum significantly altered in obesity (p < 0.05). (B) Bacteroidetes/Firmicutes ratio and relative abundance of Bacteroidetes and Firmicutes in obesity and control group (** p < 0.01, *** p < 0.001). (C) Microbial genus significantly altered in obesity (p < 0.001). The blue bar represents obesity and the red bar represents control. (D) Phage genome sizes from different hosts, with the x-axis representing the respective hosts and the y-axis indicating genome size (KB). Figure S3. Functional analysis of the microbiome. (A) Differential enrichment analysis of the KEGG modules between the obesity and control groups (LEfSe, LDA > 2.0, p < 0.001). Blue and red color represent obesity- and control- enriched modules respectively. (B) Taxon-level contribution profiles of the functional shift in control group. The x-axis depicts the ranking and statistical scores, while the y-axis represents the associated pathways. Taxa attenuating each functional shift are presented on the left side of the vertical line, whereas those driving each functional shift are depicted on the right side of the vertical line. For each KEGG pathway, the top bars represent contributions from obesity-associated taxa and the lower bars repres [file 12866_2024_3278_MOESM1_ESM.docx]

**Supplementary Figures**

**
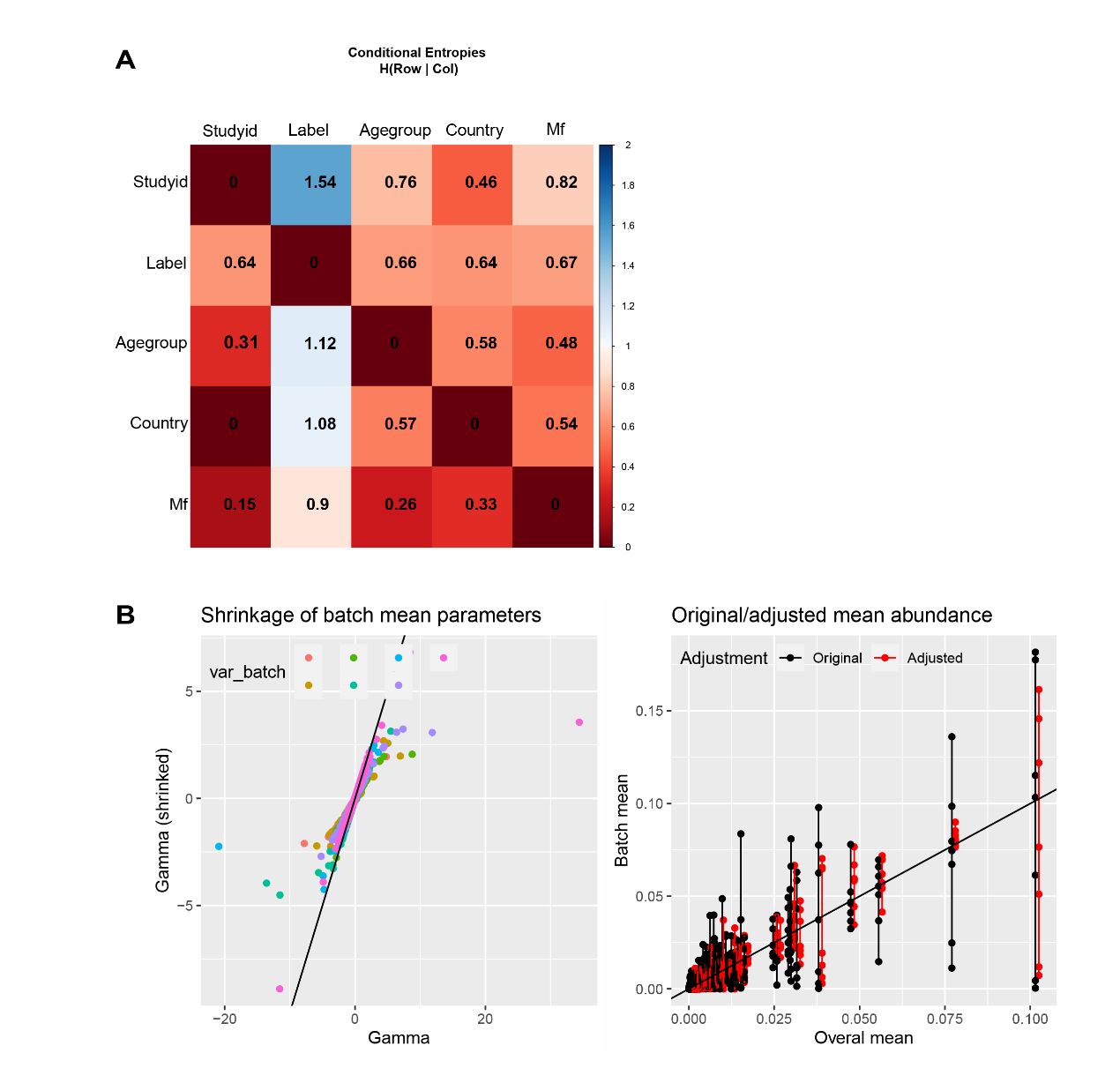
**

**Figure S1. Correction of metagenomic data from different cohorts.** (A) Assessment of confounders by SIAMCAT. (B) Scatter plot before and after batch effect correction using MMUphin. Figure (left) : Gamma parameter characterizes the mean of each feature by batch. Different batches were marked with different colors. The color points on the figure converge towards 0, indicating an increase in similarity among the parameters. Figure(right): Comparison of the mean of each feature between batch before and after adjustment. A feature is depicted as a pair of interconnected points, with each point representing that feature within a specific batch.


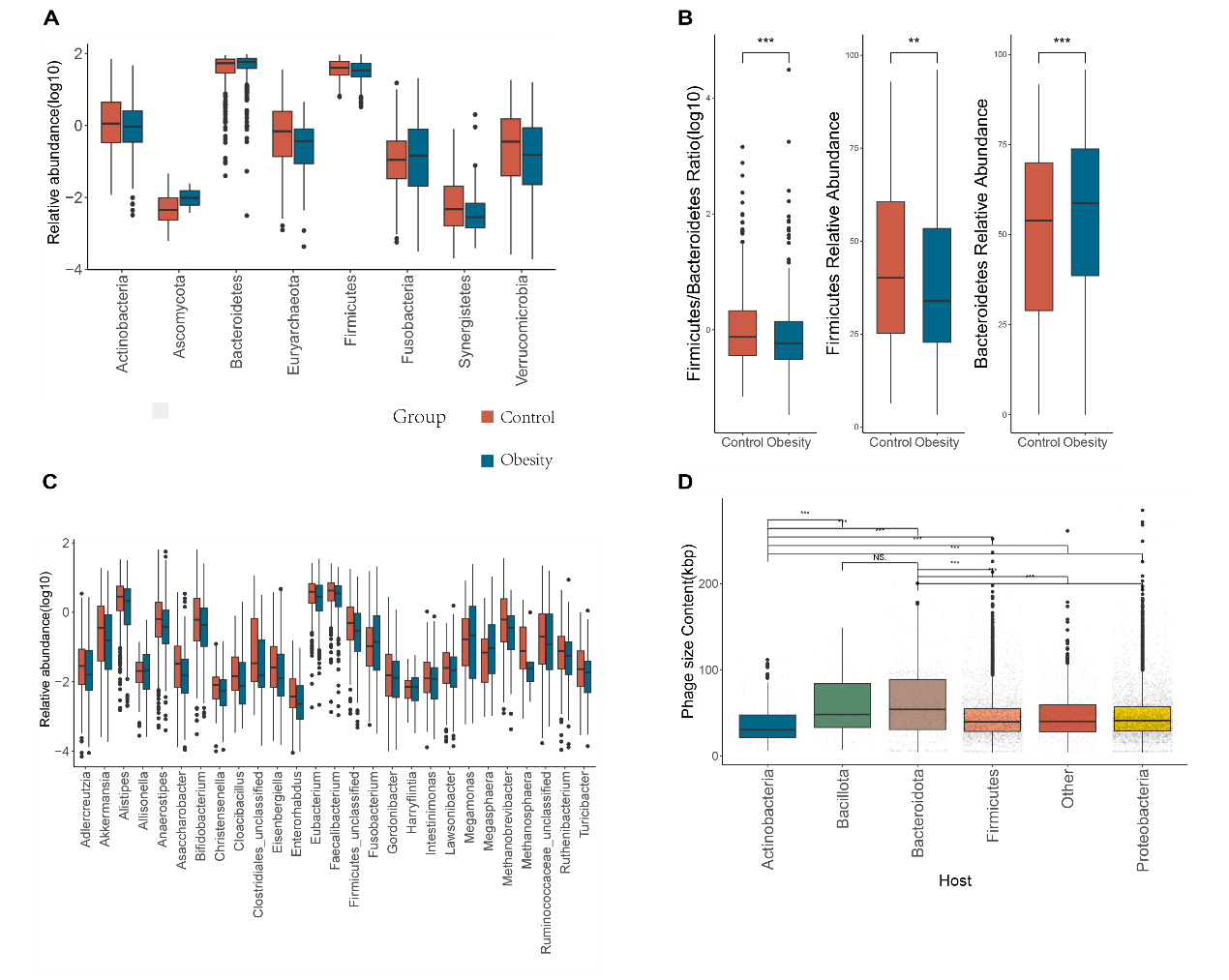


**Figure S2. Phyla displaying different abundance in obesity and viral component analysis in obesity.** (A) Bacteria phylum significantly altered in obesity (p < 0.05). (B) *Bacteroidetes/Firmicutes* ratio and relative abundance of *Bacteroidetes* and *Firmicutes* in obesity and control group (** p < 0.01, *** p < 0.001). (C) Microbial genus significantly altered in obesity (p < 0.001). The blue bar represents obesity and the red bar represents control. (D) Phage genome sizes from different hosts, with the x-axis representing the respective hosts and the y-axis indicating genome size (KB).


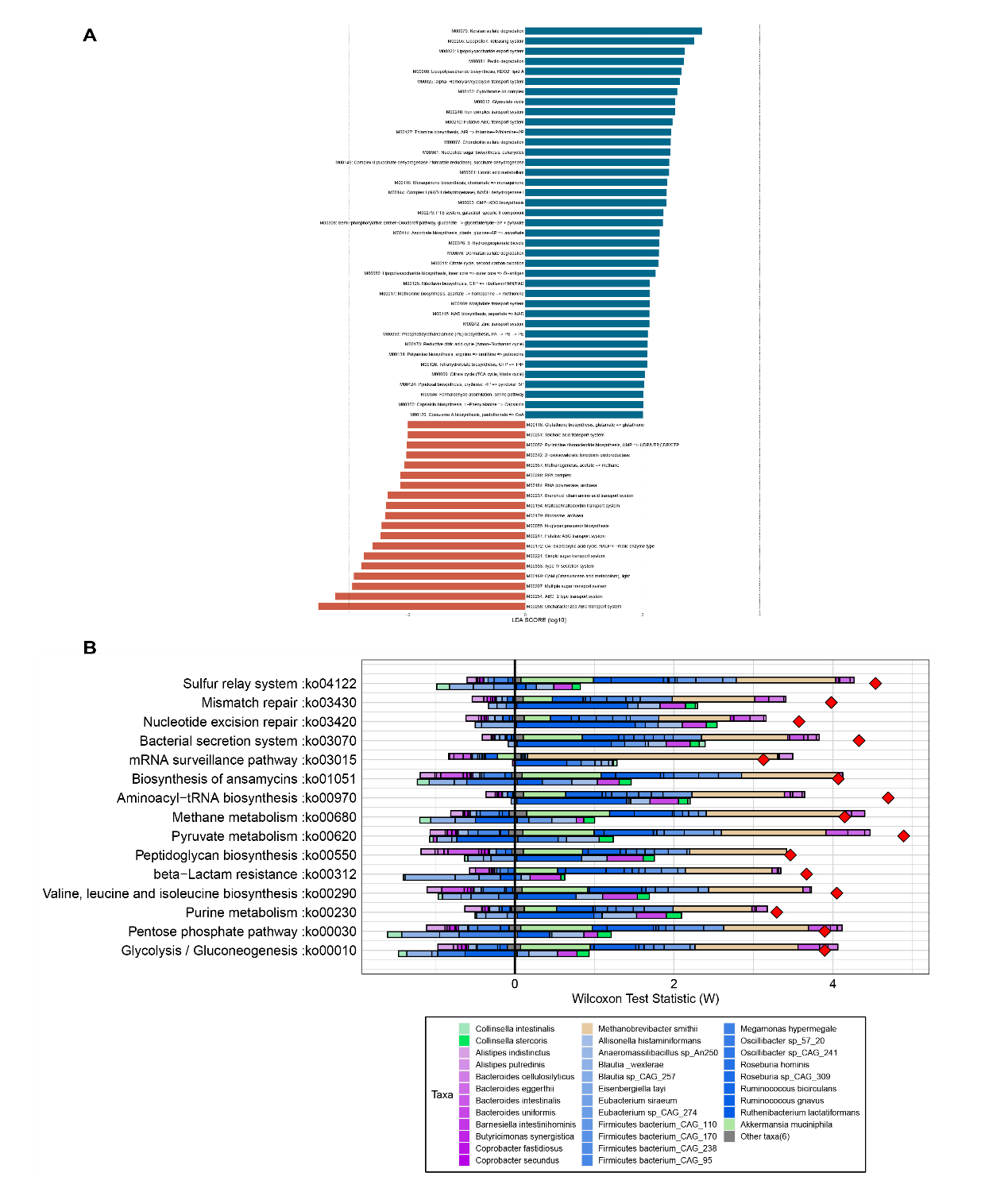


**Figure S3. Functional analysis of the microbiome.** (A) Differential enrichment analysis of the KEGG modules between the obesity and control groups (LEfSe, LDA > 2.0, p < 0.001). Blue and red color represent obesity- and control- enriched modules respectively. (B) Taxon-level contribution profiles of the functional shift in control group. The x-axis depicts the ranking and statistical scores, while the y-axis represents the associated pathways. Taxa attenuating each functional shift are presented on the left side of the vertical line, whereas those driving each functional shift are depicted on the right side of the vertical line. For each KEGG pathway, the top bars represent contributions from obesity-associated taxa and the lower bars represents contributions from obesity-depleted taxa. Red diamonds represent taxa-based functional shift scores.


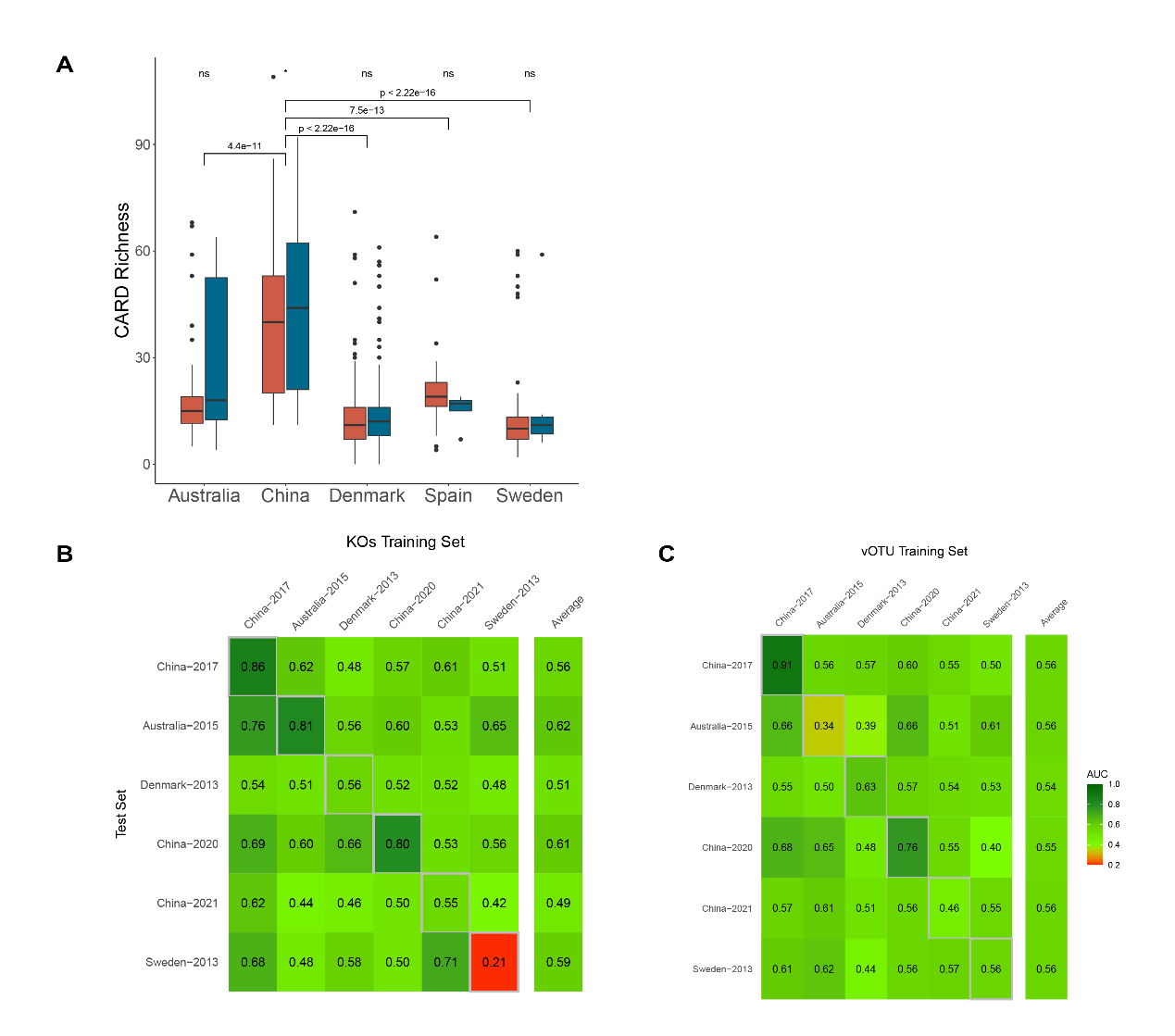


**Figure S4. The microbial ARGs distribution and cross study validation.** (A) Abundance of intestinal ARGs in populations of different countries. (B) Cross-study validation of statistical models trained on functional profiles (KEGG orthology abundances) alone. (C) Cross-study validation of statistical models trained on vOTUs abundance.
