# Supplementary material for: Integrative metagenomic analysis reveals distinct gut microbial signatures related to obesity
Source: BMC Microbiol. 2024 Apr 5;24:119. doi: 10.1186/s12866-024-03278-5 (PMC10996249; doi:10.1186/s12866-024-03278-5)
Supplement: Supplementary file 2 — Additional file 2. [file 12866_2024_3278_MOESM2_ESM.docx]

| **Table S1. Demographic and clinical details of fecal samples in each study.** | | | | | | |  |
| --- | --- | --- | --- | --- | --- | --- | --- |
| **Dataset** | **Group(Control/Obesity)** | **Age** | **BMI** | **Sex(Femal/Male)** | **Country** | **BioProject** | **PMID** |
| Australia_2015 | 35/15 | 67.28±5.77 | 26.95±3.96 | 24/26 | Australia | PRJEB7774 | 25758642 |
| China_2017 | 112/105 | Obesity:23.6±3.7 Control:23.2±1.8 | Obesity:37.026±4.69 Control:20.236±1.257 | 137/80 | China | PRJEB12123 | 28628112 |
| China_2020 | 30/32 | 31.55±10.05 | 29.29±8.99 | 34/28 | China | PRJNA597839 | 33033580 |
| China_2021 | 52/52 | Obesity:56.0±5.4 Control:54.3±6.8 | Obesity:27.7± 2.1 Control:20.8±1.5 | 49/55 | China | PRJNA686835 | 34085773 |
| Denmark_2013 | 114/164 | 50-62 | 29.71±5.99 | *156/136 | Denmark | PRJEB4336 | 23985870 |
| Spain_2014 | 54/05 | 40.44±15.16 | 24.48±3.9 | 31/28 | Spain | PRJEB1220 | 24997787 |
| Sweden_2013 | 76/16 | 70.4±0.67 | 26.36±4.2 | 92/0 | Sweden | PRJEB1786 | 23719380 |
| *Note:14 low quality data from Denmark_2013 dataset were excluded after quality control. | | | | | | |  |
